# Optimal Insertion Depth of Central Venous Catheter through the Right Internal Jugular Vein, Verified by Transesophageal Echocardiography: A Prospective Observational Study

**DOI:** 10.7150/ijms.86664

**Published:** 2024-01-01

**Authors:** Jiwon Lee, Ji-Hoon Park, Sohee Oh, Jung-Man Lee

**Affiliations:** 1 Department of Anesthesiology and Pain Medicine, Anesthesia and Pain Research Institute, Yonsei University College of Medicine, Gangnam Severance Hospital, Seoul, Republic of Korea.; 2 Department of Anesthesiology and Pain Medicine, Keimyung University School of Medicine, Keimyung University Dongsan Medical Center, Daegu, Republic of Korea.; 3 Medical Research Collaborating Center, Seoul Metropolitan Government Seoul National University Boramae Medical Center, Seoul, Republic of Korea.; 4 Department of Anesthesiology and Pain Medicine, Seoul National University College of Medicine, Seoul Metropolitan Government Seoul National University Boramae Medical Center, Seoul, Republic of Korea.; Jiwon Lee moved from 2 to 1 after completing the research.

**Keywords:** Body Height, Central Venous Catheterization, Transesophageal Echocardiography, Guideline, Jugular Veins, Superior Vena Cava

## Abstract

This prospective observational study investigated the optimal insertion depth of the central venous catheter through the right internal jugular vein using transesophageal echocardiography. After tracheal intubation, the anesthesiologist inserted a probe for esophageal echocardiography into the patient's esophagus. The investigators placed the catheter tip 2 cm above the superior edge of the crista terminalis with echocardiography, which was defined as the optimal point. We measured the inserted length of the catheter. Pearson correlation tests were performed with the measured optimal depth and some patient parameters. We made a new formula for placing the catheter at the optimal position. A total of 89 subjects were enrolled in this trial. The correlation coefficient between the measured optimal depth and the patient's parameters was the highest for patient height (0.703, p < 0.001). We made a new formula of 'height (cm)/10 - 1.5 cm'. The accuracy rate of this formula for the optimal zone was 71.9% (95% confidence interval; 62.4 - 81.4%), which was the highest among the previous formulas or guidelines when we compared. In conclusion, the central venous catheter tip was evaluated with transesophageal echocardiography, and we could make a new formula of 'height (cm)/10 - 1.5', which seemed to be better than other previous guidelines.

## Introduction

For central venous catheterization, the right internal jugular vein is most commonly used because of the advantages/disadvantages of each route 1, 2. On the other hand, it is well known that there are fatal complications due to the inadequate depth of central catheters 3-6. Therefore, numerous studies have been conducted to find the optimal depth for central venous catheterization through the right internal jugular vein. For the use of the right internal jugular vein as the route of central venous catheterization, some previous studies have presented some guidelines regarding the ideal depth based on patient height or a fixed depth 7-10. Other guidelines recommend that clinicians check the depth of the central catheter inserted, with the use of the carina on X-ray as a landmark 11. However, there has been no consensus regarding the ideal depth 12. We think that the disagreement surrounding the ideal depth is due to the indirect measurement of the ideal depth and the different methods used for measurements in each study. For example, authors in previous studies have investigated the ideal depth by various methods, such as calculating lengths using anatomical landmarks 13-15, radiologic findings 11, 16-19, cardiogenic electrical findings 20, 21, or patient height 7-9. We believe that the results of these previous studies may have been inconsistent because the previous studies used indirect methods such as simple chest radiographs.

On the other hand, until now, the most relevant factor for the ideal insertion depth in central venous catheterization in many studies and clinical practice has seemed to be considered as patient height, among the easily measurable characteristics of patients 7-9. If there was a highly accurate formula to estimate the optimal depth based on patient height, it would be a great help in actual clinical practice without image analysis.

It may be considered an ideal method to measure the optimal insertion depth of the central venous catheter while observing the catheter tip with transesophageal echocardiography (TEE) in real-time practice. However, there are absolute limitations for the routine use of TEE in central venous catheterization 22. The purpose of this study was to measure the optimal insertion depth of the central venous catheter through the right internal jugular vein using TEE in cardiac surgeries and to derive a simple meaningful formula for prediction on the basis of the measured optimal depth and patient parameters mostly correlated with the depth. The secondary aim was to compare a new simple formula for the optimal depth from our data with some guidelines introduced in previous studies.

## Methods

This prospective, observational, clinical trial was approved by the Institutional Review Board of Dongsan Medical Center (no. 2016-09-009). The study protocol was registered at clinicaltrials.gov (NCT03116724; https://clinicaltrials.gov/ct2/show/NCT03116724) before enrollment of subjects. This study was performed in accordance with the Declaration of Helsinki 2013 and written informed consent was obtained from all patients. Patients were enrolled between April 2017 and August 2017. Adult patients (age > 18) scheduled for elective open-heart surgery, requiring central venous catheterization through the right internal jugular vein and TEE, were screened for eligibility in the study. The exclusion criteria were patients who had to receive central venous catheterization at another site, such as the left internal jugular vein, subclavian vein, or femoral vein, for any reason. After we obtained informed consent from the patients, we recorded the patients' characteristics, including age, sex, height, weight, and body mass index (BMI).

First, we defined the optimal insertion depth of the central venous catheter as 2 cm above the superior edge of the crista terminalis, which was defined as the junction between the superior vena cava (SVC) and the right atrium (RA), from a review of previous studies and clinical meaning 19, 23-25. Additionally, we defined the optimal zone for the position of the tip of the central venous catheter as 1 cm above the superior edge of the crista terminalis to 3 cm above it in the study. Anesthesia and surgical techniques were performed in a standardized manner regarding the routine practice in our institute during the trial. All patients arrived in the operating room without any premedication. Patient monitoring, including electrocardiography, peripheral oxygen saturation, noninvasive blood pressure, and bispectral index began before the induction of anesthesia. After indwelling the radial artery catheter under local anesthesia with 1% lidocaine injection, general anesthesia was induced with 0.15 mg/kg midazolam, and continuous intravenous infusion of remifentanil was performed using a target concentration infusion (TCI) system. After loss of patient consciousness, 0.8 mg/kg rocuronium was intravenously administered for muscle relaxation while the patients' lungs were manually ventilated with 100% oxygen and sevoflurane. After tracheal intubation, mechanical ventilation was applied. Maintenance of anesthesia was provided with sevoflurane and continuous infusion of remifentanil. Next, a multi-plane probe (6VT-D/8.0-3.0 MHz, GE Healthcare Technologies, Wauwatosa, WI) for TEE was inserted into the patient's esophagus at the mid-esophageal level for intraoperative cardiac monitoring, and a bicaval view was obtained with control of the probe.

**Figure 1:**
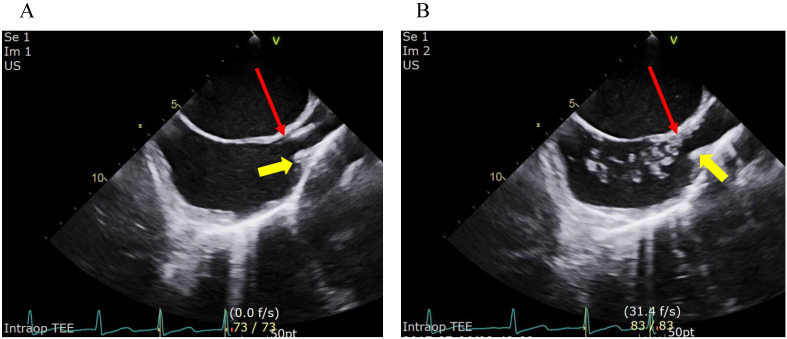
** Confirmation of the placement of the catheter tip at the superior edge of the crista terminalis on the bicaval view of transoesophageal echocardiography.** Figure 1A depicts placement of the catheter tip at the crista terminalis on the bicaval view of transoesophageal echocardiography. Figure 1B depicts the use of microbubbles to check the location of the catheter tip when the position of the tip was vague. Multiple hyperechogenic microbubbles were seen in the right atrium when the normal saline was rapidly injected through the port of the distal lumen of the central catheter. The yellow arrows indicate the superior edge of the crista terminalis. The red arrows indicate the catheter tip.

For central venous catheterization through the right jugular vein, patients were placed in the 8° Trendelenburg position by tilting the operating table and the patient's head was turned approximately 30-40° after a standard pillow was placed under the patient's right shoulder. After sterile preparation and draping of the right jugular site, an investigator inserted an introducer needle into the sterilized area to puncture the right internal jugular vein under real-time ultrasound guidance with an ultrasound machine (Vivid^TM^ S70, GE Healthcare Technologies, Wauwatosa, WI) equipped with a linear array probe (9L-D/10.0-2.4 MHz, GE Healthcare Technologies, Wauwatosa, WI). The puncture site was located at the level of the crease of the cricoid cartilage in all patients. Catheterization was performed with a 7 Fr. triple lumen catheter (Presep^®^, Edwards Lifescience, Irvine, CA) by the modified Seldinger maneuver after fresh dark blood was smoothly aspirated into the syringe, which was attached to the introducer needle. In the process of placing the central venous catheter through the right internal jugular vein while removing the guidewire, the catheter tip was first placed at the superior edge of the crista terminalis under a bicaval view of real-time transesophageal echocardiography 19, 23. The TEE examination was performed by one board-certified cardiothoracic anesthesiologist. If the catheter tip was not clearly visible, 2 to 3 ml of normal saline was quickly flushed through the catheter via the port of the distal lumen of it. Then it was easily monitored by the real-time TEE that multiple hyperechogenic microbubbles were spread into the RA from the distal tip of catheter, by which the distal tip of the catheter was identified (fig 1) 19, 26, 27. The length of the inserted part of the catheter from the skin was checked when it was finally confirmed that the catheter tip was placed at the superior edge of the crista terminalis by TEE. Next, the catheter was drawn by 2 cm to move the catheter tip to the optimal position, which was defined as 2 cm above the crista terminals in our study. The practitioner fixed the catheter by anchoring it at the skin, and a sterile dressing was placed. The final insertion length was recorded as the optimal depth for each patient. Then, anesthesia and surgery proceeded in line with routine practices in our institute.

**Figure 2:**
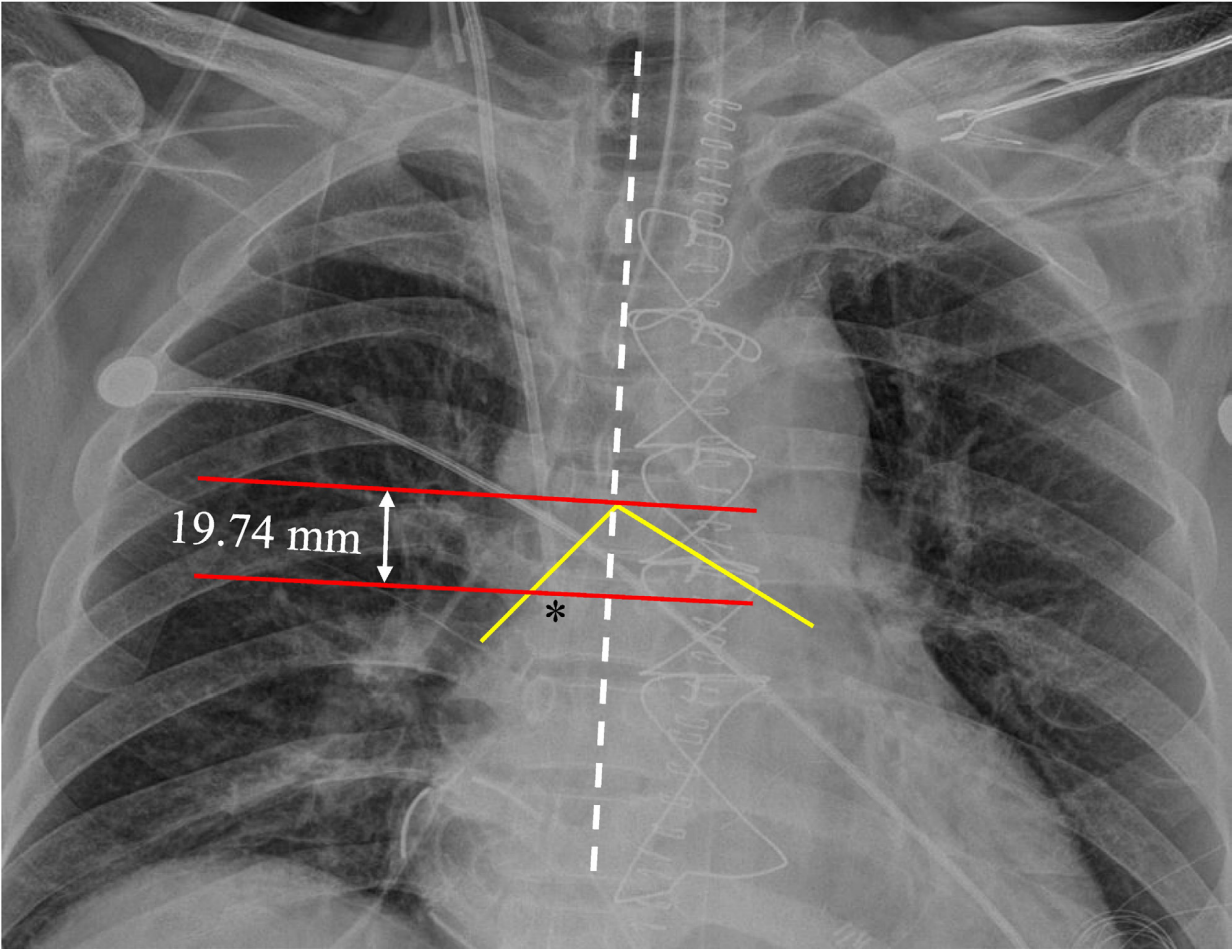
** The vertical distance from the catheter tip to the carina measured on postoperative chest X-ray.** On the day after the operation, we measured the distance from the catheter tip to the carina using the following steps. Step (1); we drew a vertical line (the white dotted line) parallel to the patient's spine. Step (2); we drew two horizontal lines at the level of the catheter tip and at the level of the carina (red lines) perpendicular to the vertical line. Step (3); we measured the vertical distance between the two horizontal lines. The intersection point of the two yellow lines represents the carina. The asterisk indicates the tip of the central venous catheter.

The primary end point was the optimal insertion depth, and we placed the catheter tip 2 cm above the superior edge of the crista terminalis using real-time TEE. The secondary endpoint was a new formula with the most correlated patient parameters to predict the optimal depth for central venous catheterization through the right internal jugular vein. Next, we calculated the accuracy of the new formula and some previous guidelines for placing the tip of the inserted catheter within the newly proposed optimal zone (from 1 cm above to 3 cm above the superior edge of the crista terminalis) in the present study and compared the accuracy rate between our new formula and the previous guidelines in terms of the placement of the central venous catheter within the optimal zone. Additionally, we evaluated the vertical distance from the catheter tip to the carina in the postoperative chest X-ray (fig 2).

When we reviewed the results of the previous study by Ahn et al. 19, the central venous catheter according to Peres' formula was located in the optimal zone, which was newly proposed by us in the present study (from 3 cm above the superior edge of the crista terminalis to 1 cm above it), in approximately 57% of patients in their study. Considering that our new formula based on the data of the present study could guide the tip of the central catheter within the newly proposed optimal zone in a higher percentage by 20% than Peres' formula in our study population, a total of 85 patients were required. Considering an ~5% drop-out rate, a total of 89 subjects were needed.

### Statistical analyses

Categorical data are presented as numbers. Continuous data are presented as the mean and standard deviation (SD). Pearson's correlation test was used to assess correlations between the real-measured optimal depth for the central venous catheter through the right internal jugular vein and the patient characteristics, including age, sex, height, weight, and BMI. In addition, we made a new simple formula for the optimal depth using the most correlated variable with the real-measured optimal depth. We calculated the predictability of a new formula for the placement of the central venous catheter within the optimal zone, which was newly proposed by us in our study population. Additionally, we calculated the predictability of some formulas or guidelines, including 'height(cm)/10' by Peres 7, 'height(cm)/10 - 1' by Czepizak et al. 8, 'height(cm)/10 - 1.3' by Lum 9, a fixed depth of 15 cm by Kim et al. 10, or 'to the carina 11. We compared the predictability of the optimal depth between our new formula and the previous formula or guidelines by using generalized estimating equation (GEE) analysis and a post- hoc test with Bonferroni correction. All data were analyzed using SPSS 21.0 (SPSS Inc. Chicago, IL, USA) and SAS 9.4 (SAS Institute Inc. Cary, NC, USA). A P-value less than 0.05 was considered statistically significant. The 95% confidence interval was calculated for adequate values.

## Results

A total of 89 patients were enrolled in this trial. Central venous catheterization was successful with the study protocol in all patients. There were no complications in the study. Table 1 shows the diagnosis and types of surgery of the 89 patients participating in the present study. The optimal depth of the catheter to the optimal point, which was 2 cm above the superior edge of the crista terminalis, was 14.5 ± 1.6 cm (95% confidence interval; 14.2 cm to 14.8 cm) under real-time ultrasound transesophageal echocardiography. The distance from the ideal depth point to the carina on postoperative chest X-ray was -1.3 cm ± 1.6 cm (95% CI; -1.0 cm to -1.7 cm); a negative value means that the catheter tip was lower than the carina. The correlation coefficients between the optimal depth and patient parameters, including sex, age, height, weight, and BMI are presented in table 2. Among the variables, patient height was most correlated with the optimal depth.

We made a simple formula, 'height (cm)/10 - 1.5 cm', to best fit the optimal zone, which was defined in our study (1-3 cm above the superior edge of the crista terminalis), on the basis of the patient's height, which was most correlated with the optimal depth of the central venous catheter tip among the patient parameters in our results.

The accuracy rate of our formula for the optimal zone of our study was calculated as 71.9% (95% confidence interval; 62.4% to 81.4%).

The accuracy rate of our formula and the previous guidelines for the optimal zone in our study population are presented in table 3. When we compared the accuracy rate with the generalized estimating equation test, there was a significant difference in guidelines, including our new formula (p<0.001). When we compared the new formula and other previous guidelines with the Bonferroni correction, the accuracy rate of our new formula was significantly higher than that of the previous guidelines except the formula suggested by Lum (table 3).

## Discussion

In our study, we verified that the patient's height was the parameter most correlated with the optimal depth of the central venous catheter tip during catheterization through the right internal jugular vein. In addition, we showed that the new formula of 'height (cm)/10 - 1.5 cm' was the best fit for locating the catheter tip within the optimal zone among various formulas or guidelines introduced in previous studies.

Many previous studies have investigated the optimal depth of the central venous catheter tip through the right internal jugular vein. However, there remains no consensus regarding the optimal depth 12. The reason may be the methodological differences between previous studies, the differences in the definition of the optimal position or optimal zone of the catheter tip, or racial differences in regards to human anatomy.

Recently, Şahinkaya et al. conducted a study to reexamine Peres' formula 28. The authors reported that when using the Peres' formula, the correct position of the catheter tip was reached in 84.7% (133/157) of cases using the right internal jugular vein as a route for central venous catheterization. Assuming that the Peres' formula was used for positioning of the catheter in our study, the 34.8% of catheters would be in the correct position in the population of the present study. It is thought that the difference in correct position of catheter tip, even though assuming the same formula, might be from that the method for identifying the optimal zone is different. We used TEE to confirm the ideal location of the catheter tip, whereas the authors used the carina on chest X-ray in the previous study 28. We thought our study had a benefit in terms of the method to identify the SVC-RA junction and the optimal point for catheter tip placement, which was defined in the present study, because of the type of surgery requiring intraoperative TEE.

Practice guidelines for central venous catheterization was released by the American Society of Anesthesiologists Task Force in 2020 29. The practice guidelines introduced three randomized controlled trials (RCTs) indicating that the intracavitary electrocardiogram (IC-ECG) method is more effective for proper placement of the central venous catheter tip. Among the three RCTs 21, 30, 31, one study with a largest sample size showed that accurate placement of the catheter tip was achieved 97% of 134 patients who received central venous catheterization using IC-ECG method through the right internal jugular vein 21. In the present study, assuming that our new formula was used, the frequency of insertion of the catheter tip within the optimal range was 72%. However, the definition of 'accurate placement' seemed to be different between the previous study and our study. In the previous study, the accurate placement might be from the middle position of the SVC to the SVC-RA junction 21. But, we defined the optimal range as from the 1 cm above the superior edge of crista terminalis, which means the SVC-RA junction, to the 3 cm above it. The optimal range defined in the present study seemed to be shorter by approximately 1 cm to the proximal direction and 1 cm to the distal direction, compared that of the previous study. If we defined the optimal range as from the middle of the SVC to the superior edge of the crista terminalis, the frequency of accurate catheter tip placement, while assuming that our new formula was used, could be calculated to 92.1% (82/89) in our study population. For only four of seven patients with the catheter tip being out of the range from the middle of the SVC to the superior edge of the crista terminalis in this study, the catheter tip would be inserted into the RA by 0.1, 01, 0.6, and 0.8 cm, respectively. From this, we believe that our new formula might be an easy, safe, and accurate method for proper placement of central catheter tip for access through the right internal jugular vein, even though it might not ensure accurate positioning of the catheter tip nearly as close to 100% as in the IC-ECG method 21, and no information about the catheter tip position is available while clinicians is placing the catheter with the use of it. Formula based on the patient height, such as our new formula cannot be useful in order to detect primary malposition during insertion procedure. In addition, the formula based on the patient height cannot be accurate if venous puncture is at a higher or lower position, even though the puncture site was fixed at the crease of the level of the cricoid cartilage in the present study. Further studies must be needed to investigate clearly difference in accurate positioning of the catheter tip between different methods, including our new formula and the IC-ECG method.

We did not present a new formula for the optimal depth on the basis of real-time TEE measurements but on the patient's height. After a review of previous studies on this issue, we predicted that the patient's height among patient's anatomical characteristics would be a main correlating factor for the optimal depth 7-9. We hoped that the new formula from our data would be simple for use in every clinical situation. Some previous studies used some measures from chest X-ray, which seems to be very simple to use. However, central venous catheterization should be performed abruptly without chest X-ray in some emergent situations. Additionally, chest X-ray during pre-anesthesia evaluation is not mandatory in all healthy and young patients in some countries.

Another possible reason for the controversy in determining the optimal depth may be the difference in the defined optimal position or optimal zone for the placement of the central catheter tip in each study. The US Food and Drug Administration recommends that the catheter should not be located in or allowed to migrate into the heart 12. Some previous studies showed that the mean SVC length was approximately 7 cm 10, 32. Some previous studies recommended that the catheter tip should be above the pericardial reflection 11, 33. However, the upper margin of the pericardial reflection could be up to 5 cm above the SVC-RA junction in some people 11, and catheter tip placement more than 4 cm above the SVC-RA junction can lead to catheter malfunction 34. In a recent study, moreover, the authors argued that free-floating catheter tips should be more important than placement above the pericardial reflection 19. Finally, some previous studies suggested that the optimal position of the catheter tip should be 2 cm above the atrio-caval junction 5, 35, 36. From this review, we defined the optimal point of the catheter tip as 2 cm above the superior edge of the crista terminalis, and we defined the optimal zone as within 3 cm above to 1 cm above the superior edge of the crista terminalis with a safety margin of 1 cm from the optimal point.

It may be true that there are racial differences in human anatomy. We investigated the optimal depth of the central venous catheter through the right internal jugular vein in Korean only. To verify our results in many races or to determine a new simple formula or guideline to fit all humans regardless of race, multiple studies or international multi-center studies are needed.

There were also some studies comparing the accuracy of different guidelines from previous studies for the ideal depth of central venous catheterization due to this discrepancy 19, 31, 37. We also compared the accuracy of our new formula and that of the previous guidelines. For the optimal zone, which was defined in our study, the accuracy was higher with our new formula than with some previous guidelines except one study by Lum, which was similar to ours. We performed this trial with a sample size of 89. Considering that there are numerous performances of central venous catheterization, further studies with large sample sizes should be performed to verify whether our new formula is superior.

There are some limitations of our study. First, we defined the optimal zone as to 1-3 cm above the superior edge of the crista terminalis. This could not be absolute or based on any strong evidence. Even though the optimal point of our study could be below the pericardial reflection, we agreed with the concept of the study of Ahn et al. 19, that is, the catheter could float parallel to the vascular wall of the SVC if the catheter tip was placed at its lower part. However, we believe that the catheter tip should not be placed in the cardiac chamber. Therefore, we defined the optimal zone for the catheter tip similarly in our study. Additionally, we speculate that the reason why perforation of the SVC by the central catheter tip has rarely been reported or has not been reported in these days is due to the advancement of the material and the design of the catheter tip 38. Second, we performed this study in 89 patients receiving cardiac surgery according to various diagnosis, which was shown in table 1, because of ethical reason in the use of transesophageal echocardiography. The morphology of heart and great vessels of them may have been changed from the progress of the cardiac diseases diagnosed. Therefore, there could be limitations in applying the results of this study to patients without cardiac disease. However, we believe that the morphological change in the part from the internal jugular vein to the end of SVC is relatively minimal. Third, we calculated the sample size from the result of the study of Ahn et al. 19. Our sample size seemed to be small for applying our new formula in the general population, considering that there could be anatomical differences between persons and races.

In conclusion, we evaluated the optimal depth of the central venous catheter through the right jugular vein with real-time TEE. The optimal depth was most correlated with patient height, and we found a new formula of 'height (cm)/10 - 1.5' for the optimal depth, which was better than other guidelines in our study population.

## Data availability statement

The datasets generated during and/or analyzed during the current study are available from the corresponding author on reasonable request.

## Author contributions

Substantial contributions to the conception or design of the work: JL, JML.

Acquisition, analysis of data for the work: JL, SO, JHP.

Drafting the work: JL, JML.

Final approval of the version: JL, JHP, SO, JML.

Agreement to be accountable for all aspects of the work in ensuring: JL, JHP, SO, JML.

## Figures and Tables

**Table 1:** Diagnosis and type of surgery for the study population

Diagnosis	Type of surgery	Number
Coronary artery disease (CAD)	Coronary artery bypass graft (CABG)	31
Aortic aneurysm (AA)	AA repair	5
Mitral stenosis or regurgitation (MS/MR)	Mitral valve replacement (or repair)	14
Aortic stenosis or regurgitation (AS/AR)	Aortic valve replacement (AVR)	20
Tricuspid regurgitation	Tricuspid valve repair	3
Atrial septal defect (ASD)	ASD closure	2
AA + AS or AR	Bentall operation	5
Cardiac tumor	Tumor removal	2
Dual valvular disease	Dual valve replacement (or repair)	4
AS + CAD	CABG + AVR	2
Idiopathic hypertrophic subaortic stenosis	Septal myomectomy	1

**Table 2:** Demographic characteristics for the study patients and correlation coefficient between the optimal depth of central venous catheter and the patient characteristics

Characteristics	(n = 89)	Coefficient	P-value
Sex (M/F, n)	51/38	-0.591	< 0.001
Age (y)	66.1 ± 14.5	-0.180	0.092
Height (cm)	160.4 ± 9.3	0.703	< 0.001
Weight (kg)	61.7 ± 11.3	0.550	< 0.001
BMI (kg/m^2^)	23.8 ± 3.0	0.166	0.121

Data except sex were presented as mean ± standard deviation. Sex was presented as numbers of patients. BMI: body mass index

**Table 3:** Accuracy rate of new formula and other previous guidelines for placing the central venous catheter in the optimal zone, which was defined between 3 cm above the superior edge of the crista terminalis and 1 cm above it.

Guidelines	Equation (cm)	Accuracy rate (95% CI)	OR (95% CI)	P-value
New formula*	H/10 - 1.5	71.9% (62.4-81.4)		
Peres 7	H/10	34.8% (24.7-44.9)	4.79 (2.53, 9.09)	< 0.001
Czepizak et al. 8	H/10 - 1	58.4% (48.0-68.9)	1.82 (1.25, 2.66)	0.002
Lum 9	H/10 - 1.3	67.4% (57.5-77.3)	1.24 (0.92, 1.66)	0.153
Kim et al. 10	15	50.6% (40.0-61.2)	2.50 (1.45, 4.32)	0.001
The carina 11	to the carina on the CXR	36.0% (25.8-46.1)	4.56 (2.38, 8.73)	< 0.001

CI; confidence interval, OR; odds ratio when the new formula*, which was suggested from our results, was compared with the others as reference, H; patient's height (cm), CXR; chest X-ray after the surgeries, P-value was presented as the Bonferroni corrected P-value after generalized estimating equations (GEE) analysis.
